# Influence of Intratumor Microbiome on Clinical Outcome and Immune Processes in Prostate Cancer

**DOI:** 10.3390/cancers12092524

**Published:** 2020-09-05

**Authors:** Jiayan Ma, Aditi Gnanasekar, Abby Lee, Wei Tse Li, Martin Haas, Jessica Wang-Rodriguez, Eric Y. Chang, Mahadevan Rajasekaran, Weg M. Ongkeko

**Affiliations:** 1 Department of Surgery, Division of Otolaryngology-Head and Neck Surgery, UC San Diego School of Medicine, San Diego, CA 92093, USA; jim095@ucsd.edu (J.M.); agnanase@ucsd.edu (A.G.); acl008@ucsd.edu (A.L.); wtl008@ucsd.edu (W.T.L.); 2 Research Service, VA San Diego Healthcare System, San Diego, CA 92093, USA; 3 Moores UCSD Cancer Center, University of California San Diego, La Jolla, CA 92093, USA; mhaas@ucsd.edu; 4 Division of Biological Sciences, University of California San Diego, La Jolla, CA 92093, USA; 5 Department of Pathology, University of California, San Diego, CA 92093, USA; Jessica.Wang-Rodriguez@va.gov; 6 Pathology Service, VA San Diego Healthcare System, San Diego, CA 92161, USA; 7 Department of Radiology, University of California, San Diego, CA 92093, USA; e8chang@health.ucsd.edu; 8 Radiology Service, VA San Diego Healthcare System, San Diego, CA 92161, USA; 9 Department of Urology, University of California San Diego, La Jolla, CA 92093, USA; mrajasekaran@ucsd.edu; 10 Urology Service, VA San Diego Healthcare System, San Diego, CA 92161, USA

**Keywords:** prostate cancer, intratumor microbiome, immune processes

## Abstract

**Simple Summary:**

While the intratumor microbiome has been largely unexplored in relation to prostate cancer development, our research shows that microbes may play an anti-tumor or pro-tumor role to significantly alter clinical course in prostate cancer patients. We found that the presence and absence of specific microbes are strongly correlated with known biomarkers of prostate cancer, including increased androgen receptor expression, prostate-specific antigen level, immune-associated gene dysregulation, stem-cell related gene overexpression, cancer pathways, and known chromosomal alterations. Our results provide important insight on potential mechanisms by which intratumor microbes may greatly contribute to prostate cancer progression and prognosis. We hope our results can be validated in future studies, and the key microbes that we identified can be used as effective targets for more specialized prebiotic and probiotic treatments for prostate cancer.

**Abstract:**

Although 1 in 9 American men will receive a diagnosis of prostate cancer (PC), most men with this diagnosis will not die from it, as most PCs are indolent. However, there is a subset of patients in which the once-indolent PC becomes metastatic and eventually, fatal. In this study, we analyzed microbial compositions of intratumor bacteria in PC to determine the influence of the microbiome on metastatic growth. Using large-scale RNA-sequencing data and corresponding clinical data, we correlated the abundance of microbes to immune pathways and PC risk factors, identifying specific microbes that either significantly deter or contribute to cancer aggressiveness. Interestingly, most of the microbes we found appeared to play anti-tumor roles in PC. Since these anti-tumor microbes were overrepresented in tumor samples, we believe that microbes thrive in the tumor microenvironment, outcompete cancer cells, and directly mitigate tumor growth by recruiting immune cells. These include *Listeria monocytogenes*, *Methylobacterium radiotolerans JCM 2831*, *Xanthomonas albilineans GPE PC73*, and *Bradyrhizobium japonicum*, which are negatively correlated with Gleason score, Tumor-Node-Metastasis (TNM) stage, prostate-specific antigen (PSA) level, and Androgen Receptor (AR) expression, respectively. We also identified microbes that contribute to tumor growth and are positively correlated with genomic alterations, dysregulated immune-associated (IA) genes, and prostate cancer stem cells (PCSC) genes.

## 1. Introduction

Prostate cancer (PC) is the most commonly diagnosed cancer among U.S. males and currently ranks second in cancer mortality with over 33,000 deaths estimated this year [1]. Although most PCs are indolent with a 5-year survival rate of 100%, the subset of PCs that become metastatic only have a 5-year survival rate of 31%. In fact, metastasis accounts for more than 90% of all cancer-associated deaths [2]. Current treatment options for metastatic castration-resistant PCs can only marginally prolong the median overall survival (OS). For example, docetaxel-based therapy prolongs OS for just 3 months, and Androgen Receptor (AR) antagonist enzalutamide showed only a 5-month survival advantage in patients with metastatic PC [3,4]. Thus, identifying the main causes for metastasis, along with controlling metastasis, continue to be the primary challenges in PC. While the origin of PC is not definitively known, prior findings seem to support the notion that prostate cancer stem cells (PCSC) may initiate metastasis and PC progression [5]. In order for epithelial cancer cells to leave the primary site and arrive at secondary organs, they must undergo epithelial–mesenchymal transition (EMT). Once the cancer cells have entered the bloodstream, crossed vessel walls, and settled into the target organ, they must reverse back to their epithelial status through mesenchymal epithelial transition (MET) to grow into metastatic tumors (MET). These sequential transitions between EMT and MET are driven by cell plasticity, which is a characteristic property of cancer stem cells [6]. However, although overwhelming evidence supports the role of PCSCs in PC initiation, it is still uncertain what factors lead to the acquisition of plasticity, which allows cancer stem cells to evolve and promote a metastatic phenotype. 

The human microbiome consists of a collection of microorganisms inside and on the surface of the human body. All humans share a “core” microbiota [7], but differences in diet and host phenotype can influence the human microbiome [8], making the human microbiota ecosystem taxonomically diverse [9]. These microbes play both pro-tumor and anti-tumor roles through a variety of mechanisms [10]. Such mechanisms include modulating the body’s immune response, as well as producing toxins and metabolites that cause inflammation or damage host cell DNA [11,12,13]. Recent research on the human microbiome has demonstrated the increasingly significant role of microbes in cancer pathogenesis [6,14,15]. A differential abundance of microbes in the gut has been most heavily investigated, and studies have found that changes in gut microbial composition affect the development of colorectal cancer [16]. Studies have shown that bacteria present in the breasts, lungs, bladder, pancreas, and prostate are also associated with cancer, although these associations are limited by sample size [15,17,18,19,20,21,22,23,24]. It has been found that microbes may influence the initiation and progression of PC via direct interactions at the site of the cancer development, such as bacterial prostatitis, and indirect interactions, including the mediation of the immune response [25]. In prostate cancer lesions, varied bacterial populations enhance the pro-inflammatory response, contributing to cancer development [26]. For example, analysis of the microbial ecosystem of tumoral, peritumoral, and non-tumoral prostate tissue collected after radical prostatectomy found *Enterobacteriaceae* exclusively in tumoral samples [26]. *Enterobacteriaceae* is known to be more abundant in inflammatory and neoplastic conditions [26,27]. In addition to contributing to prostatic inflammation, which is a risk factor for prostate cancer development, microbes have also been linked to response to treatment [28,29,30]. For instance, Sfanos et. al observed a unique microbial signature in patients undergoing oral androgen deprivation therapy (ADT) that was absent in prostate cancer patients not on ADT, suggesting a possible association between androgen levels and the human microbiome [31]. Based on recent findings, we believe that the prostate microbiome may possess a significant role in the PC metastasis, the regulation of PCSC gene expression, and a vital regulator of known PC risk factors, including elevated prostate-specific antigen (PSA) and androgen levels. 

In this study, we characterized the presence of microbes in prostate cancer using sequencing data from 242 PC patients accessed through The Cancer Genome Atlas (TCGA). This large number of patients allows us to stratify patients based on clinical variables and provides power to our statistical analyses. We conducted differential abundance analysis to identify the microbes that are most significantly dysregulated in PC. We investigated the correlation of microbial presence to clinical variables, including Gleason score, pathologic stage, and PSA levels. We measured the association between microbe abundance and inflammation by correlating microbial presence with genomic alterations, immune-associated genes, and tumor infiltrating immune cells. In addition, we correlated microbial presence with the expression of the Androgen Receptor, and we studied the correlations between microbial abundance and stem cell gene expression. We observed that patient data were exclusively from the United States and Germany, and since this geographic region is known to influence the microbiome, we also compared the microbiome composition between these two locations. Specifically, we conducted Qiime diversity calculations and compared microbe composition in patients between the two countries. With this study, we aim to provide an atlas of microbial abundance and its clinical significance in PC initiation and progression. 

## 2. Results

### 2.1. Data Acquisition

**Figure 1:**
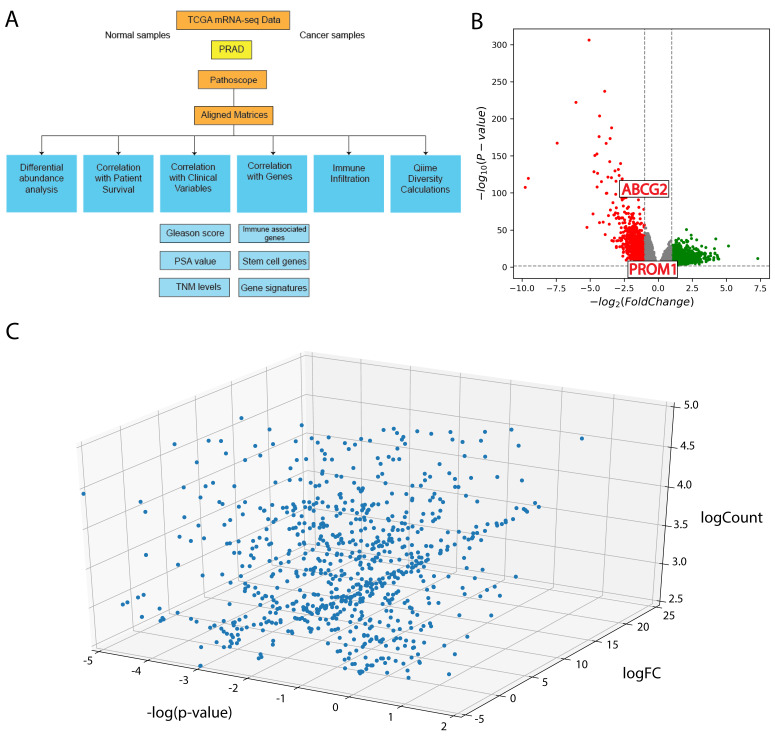
(**A**) Schematic of project analyses (**B**) Volcano plot illustrating differentially expressed genes in prostate cancer (PC). Points outside the gray area and grid lines have significant log fold change (logFC). (**C**) 3D plot illustrating differential abundance of microbes in PC.

RNA-sequencing data were downloaded from TCGA for prostate adenocarcinoma (PRAD). Since RNA-sequencing captures RNA of bacterial origin and RNA from human cells, we used the direct alignment tool Pathoscope to align non-human RNA-sequencing reads to sequenced bacterial genomes deposited in the National Center for Biotechnology Information (NCBI) nucleotides database (Figure 1A). 

### 2.2. Differential Expression of Genes and Differential Abundance of Microbes 

A total of 12,281 genes were significantly dysregulated in PC compared to adjacent normal samples. (Figure 1B). In further analyses, we specifically studied dysregulated IA genes and PCSC genes. 

### 2.3. Comparison between Microbial Abundance of Tumor Samples and Available Adjacent Normal Samples 

We found the log fold change of the majority of microbes to be positive, and more than half of the microbes had a magnitude of log fold change greater than 1, indicating that the microbes are more highly expressed in PC samples than in normal samples (Figure 1C). We also concluded that a high microbe presence in tumor samples is not a result of contamination, since contaminant microbes would have a log fold change of 0. Contaminant microbes would not be consistently overrepresented in cancer samples compared with in normal samples, when tumor tissue and adjacent normal tissue follow the same procedure before and during sequencing. 

### 2.4. Contamination Correction

To verify true tumor microbe presence, excluding microbial influence from sequencing centers, laboratory equipment, and hospitals, we conducted two methods of contamination correction to detect microbial contaminants. We identified six potential contaminants: *Capnocytophaga canimorsus*, *Propionigenium maris*, *Xanthomonas citri*, *Agrobacterium*, *Mycoplasma mycoides*, and *Cellvibrio japonicus Ueda 107*. Among these, *Agrobacterium and Propionigenium maris* are known as agents of nosocomial infections [32,33,34]. We eliminated these microbial contaminants from subsequent analyses. 

### 2.5. Microbe Correlation with Clinical Variables

By investigating the correlation of microbes with patients’ Gleason Scores, Tumor-Node-Metastasis (TNM) stages, and PSA values, we examined the relationship between microbe abundance and prostate cancer aggressiveness.

#### 2.5.1. Gleason Scores

**Figure 2:**
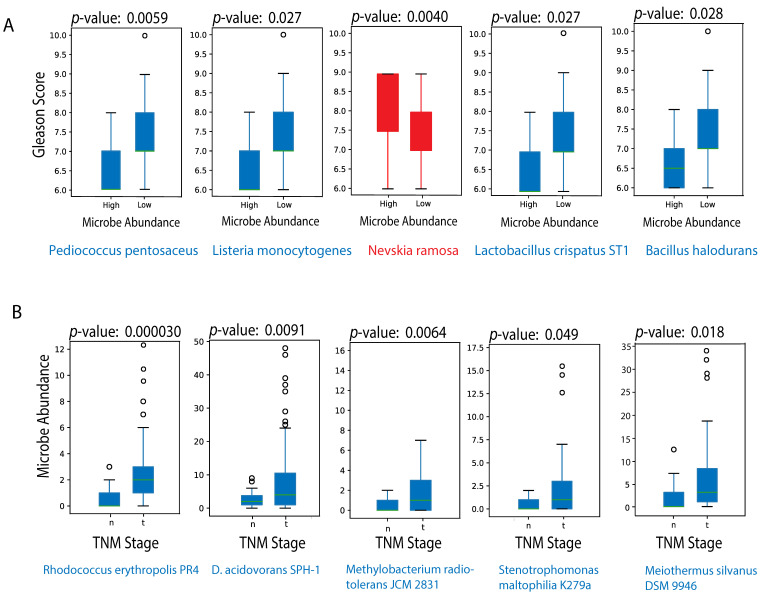
(**A**) Microbes significantly correlated with Gleason score. Microbe abundance was converted to a binary variable of “High” and “Low”, as labelled on the x-axis. The y-axis corresponds to Gleason score, which was defined as a continuous variable. The blue boxplots show negative correlations, and the red boxplots show positive correlations. (**B**) Microbes significantly correlated Tumor-Node-Metastasis (TNM) cancer stage. Microbe abundance was kept as a continuous variable of counts and plotted on the y-axis. The x-axis corresponds to TNM stage. The microbes we identified to be most correlated with TNM classification were all negatively correlated.

We found that *Pediococcus pentosaceus* (*p* = 0.0059), *Listeria monocytogenes* (*p* = 0.027), *Lactobacillus crispatus ST1* (*p* = 0.027), and *Bacillus halodurans* (*p* = 0.028) are strongly negatively correlated with patients’ Gleason scores. On the other hand, we found that *Nevskia ramosa* (*p* = 0.0040) is strongly positively correlated with Gleason scores (Figure 2A). Of the four microbes we identified as negatively correlated with Gleason scores, three have previously been shown to have anticancer properties. A certain strain of *Pediococcus pentosaceus* has been shown to mitigate colon cancer development by producing conjugated linoleic acid that triggers apoptosis for cancer colonocytes in vivo, and *Listeria monocytogenes* (*Lm*) has been found to stimulate robust innate and adaptive immunity that is crucial for an effective anti-tumor response [35,36]. Pathogen-associated molecular patterns (PAMPs) from *L. monocytogenes* are recognized by cell surface Toll-like receptors on macrophages and dendritic cells, resulting in immune activation and the release of pro-inflammatory cytokines [37]. More importantly, *Lm* is able to escape phagosomes and enter the cytosol through the secretion of sulfhydryl-activated hemolysin listeriolysin [38]. Upon entering the cytosol, *Lm* can secrete protein antigens that trigger the induction of *Lm*-specific cytotoxic T-cell lymphocytic responses [39]. In addition to triggering a robust immune response, *Lm* has been shown to increase the production of intracellular reactive oxygen species (ROS) and apoptotic cell death, highlighting the anticancer properties of the bacteria [40,41]. Our results are also consistent with existing literature that shows certain amounts of *Lactobacillus crispatus* cause a reduction in tumor size and lowered Cox2 expression in tumor tissues in an experimental model of breast cancer in mice [42]. The final microbe negatively correlated with Gleason scores, *Bacillus halodurans*, has not previously been associated with cancer. On the other hand, we found that *Nevskia ramosa* (*p* = 0.0040) is positively correlated with Gleason scores. *Nevskia ramosa* has not previously been associated with cancer. Further research is needed to investigate the role of *Bacillus halodurans* and *Nevskia ramosa* in prostate cancer pathogenesis.

#### 2.5.2. TNM Stages

We also found that *Rhodococcus erythropolis PR4* (*p* = 0.000030), *Delftia acidovorans SPH-1* (*p* = 0.0091), *Methylobacterium radiotolerans JCM 2831* (*p* = 0.0064), *Stenotrophomonas maltophilia K279a* (*p* = 0.049), and *Meiothermus silvanus DSM 9946* (*p* = 0.018) are negatively correlated with TNM cancer staging. No microbes were found to be positively correlated with TNM staging (Figure 2B). Of the five microbes we identified as negatively correlated with TNM staging, two strains, *Delftia acidovorans SPH-1* and *Meiothermus silvanus DSM 9946*, had not been identified in clinical cases previously. While the remaining three strains have not previously been associated with cancer, they are often observed either in immunosuppressed patients or in patients with in-dwelling intravascular devices. Immunosuppression appears to be a major risk factor in the pathogenesis of *Rhodococcus* infections, and cases of *Methylobacterium radiotolerans* infection in cancer patients have mostly been associated with immunocompromised patients with long-indwelling central venous catheters [43,44]. Similarly, *Stenotrophomonas maltophilia* in patients with cancer are associated with infected in-dwelling intravascular devices [45,46,47]. Further research is needed to explain the negative correlation between TNM staging and these microbes.

#### 2.5.3. PSA Values

**Figure 3:**
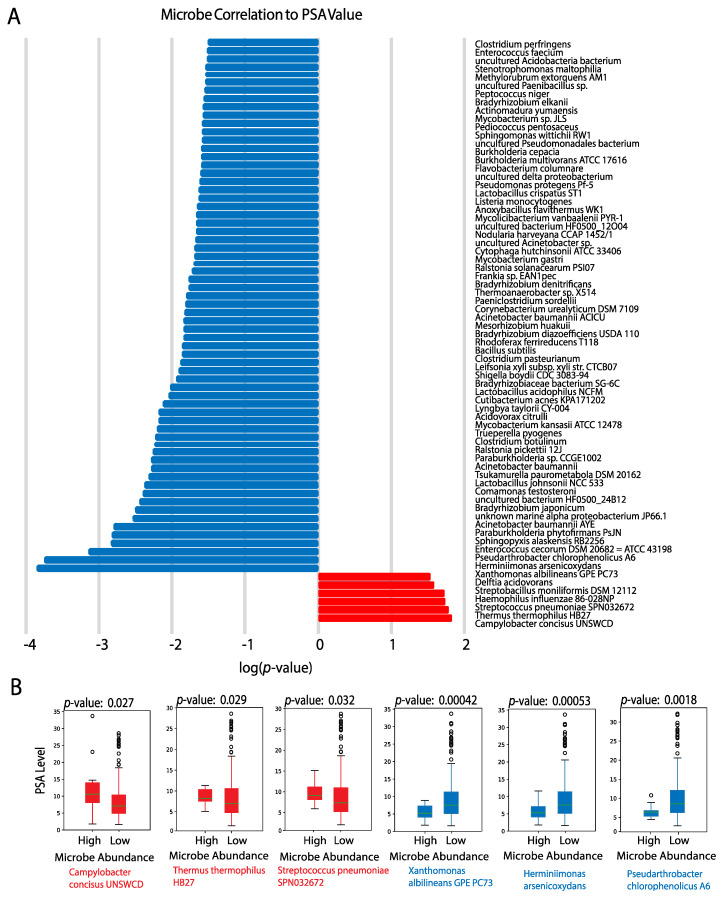
(**A**) Microbes significantly correlated with prostate-specific antigen (PSA) (*p* < 0.05). Red bars indicate positively correlated microbes, and blue bars indicate negatively correlated microbes. (**B**) Select boxplots of microbes that are most significantly correlated with PSA level. Microbe abundance was converted to a binary variable of “High” and “Low” as labeled on the x-axis. The y-axis corresponds to PSA level, which was defined as a continuous variable.

Lastly, we found 234 microbes to be significantly associated with elevated PSA levels (*p*-value < 0.05) (Figure 3A). Microbes with the strongest correlations between their abundance in tumor samples and PSA value include *Campylobacter concisus UNSWCD* (*p* = 0.027), *Thermus thermophilus HB27* (*p* = 0.029), and *Streptococcus pneumoniae SPN032672* (*p* = 0.032), which are positively correlated with PSA value. On the other hand, *Xanthomonas albilineans GPE PC73* (*p* = 0.00042), *Herminiimonas arsenicoxydans* (*p* = 0.00053), and *Pseudarthrobacter chlorophenolicus A6* (*p* = 0.0018) were observed to be strongly negatively correlated with PSA value (Figure 3B). Of the three microbes we found to be positively correlated with PSA values, one had an unanticipated positive correlation with PSA values. *Thermus thermophilus* produces L-asparaginase, an enzyme that converts L-asparagine to ammonia and L-aspartic acid [48]. Purified L-asparaginase from *Thermus thermophilus* has shown to selectively inhibit the growth of several cancers [49]. Although its effect has not been investigated in prostate cancer, existing literature shows that asparagine synthetase is a target in castration-resistant prostate cancer, and prostate cancer cells are especially sensitive to arginine deprivation because they lack argininosuccinate synthetase (ASS), the rate-limiting biosynthetic enzyme responsible for intracellular arginine synthesis [50,51]. Further research must be done to explain the unexpected positive correlation between *Thermus thermophilus HB27* and PSA values. This could involve exploring the relationship between *Thermus thermophilus* and the degradation of PSA. Another possible reason for the unexpected correlation is the lowered enzymatic activity of L-asparaginase. L-asparaginase presents optimal activity at pH 9.2, but the pH of prostatic fluid ranges from 6.1 to 6.5. While there is a paucity of research on the association between prostate cancer and the remaining two microbes, these microbes are associated with esophageal adenocarcinoma (EAC). *Campylobacter concisus* induces mucosal inflammation, as the presence of *C. concisus* in the esophagus is associated with a marked increase in pro-inflammatory cytokines, while depletion of *Streptococcus pneumoniae* in the oral microbiome was found to be associated with a lower esophageal adenocarcinoma risk [52]. However, further research is needed to establish causal relationships between *Streptococcus pneumoniae* and *Campylobacter concisus* and prostate cancer. The microbes we found negatively correlated with PSA values had not been previously identified in humans. 

### 2.6. Correlation to Immune-Associated Processes

#### 2.6.1. Correlation to Genomic Alterations (REVEALER)

**Figure 4:**
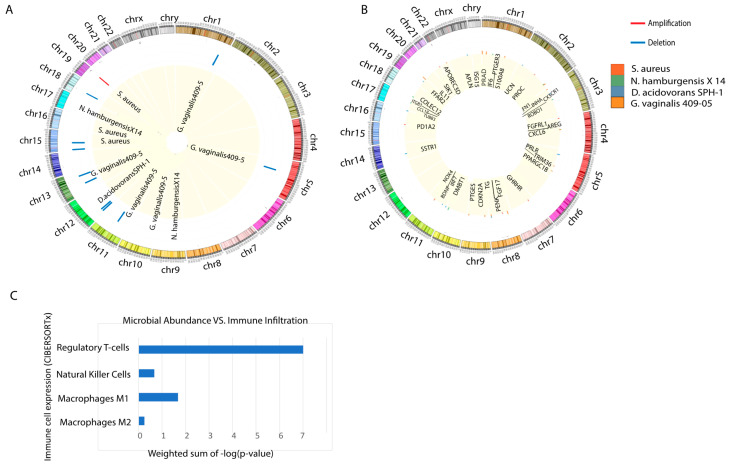
(**A**) Summary of loci for genomic alterations that are associated with microbe abundance (REVEALER, |CIC| > 0.3). (**B**) Correlation between abundance of microbes that are significantly associated with genomic alterations, identified using REVEALER, with immune-associated (IA) gene expression. A bar is plotted at each IA gene’s genomic locus. The bar color represents microbe identity and the bar height corresponds to the magnitude of correlation. (**C**) Correlation between microbial abundance and immune infiltration. The Kruskal–Wallis test was employed to determine the p-value of correlation between individual microbes and different immune cell populations. −log(*p*-value) was calculated and added together to determine the weighted sum.

Using the REVEALER program, we examined the correlation between mutations and copy number alterations with microbe abundance (Figure 4A). We found *Delftia acidovorans SPH-1*, *Gardnerella vaginalis 409-05*, *Nitrobacter hamburgensis X14*, and *Staphylococcus aureus* to be the most significantly correlated microbes with genomic alterations. Among the 11 strongest correlations, *S. aureus* is the only microbe correlated with an amplification in chromosome 19, and it is also correlated with two deletions in chromosome 15. *D. acidovorans SPH-1*, *G. vaginalis 409-05*, and *N. hamburgensisX14* are all correlated with deletions, with *G. vaginalis 409-05* alone was associated with five deletions. To further investigate how the tissue microbiome may contribute to transformation-related genomic alterations, or how genomic alterations could occur first and then trigger changes in microbiome composition, we correlated *D. acidovorans SPH-1*, *G. vaginalis 409-05*, *N. hamburgensisX14*, and *S. aureus* with immune-associated gene dysregulation. 

#### 2.6.2. Correlation to Immune-Associated (IA) Genes

A potential consequence of the strong correlations between microbe abundance and genomic alterations is that the latter could produce a change in immune-associated gene expression at the alteration site. Therefore, we plotted the location of dysregulated immune-associated genes associated with the specific microbes found to correlate with genomic alterations (Figure 4B). We found *G. vaginalis*, the microbe that was significantly associated with the greatest number of deletions, to also be significantly correlated with the greatest number of dysregulated IA genes. *G. vaginalis* was associated 15 dysregulated IA genes, *N. hamburgensisX14* was associated with 14 dysregulated IA genes, *D. acidovorans SPH-1* was associated with 11 dysregulated IA genes, and *S. aureus* was associated with 7 dysregulated IA genes. While some of the locations of the dysregulated IA genes do correspond to the alteration sites associated with each microbe, there was no direct correlation or pattern between the location of dysregulated IA genes and the site of genomic alterations associated with each microbe. We found PC microbe expression to significantly correlate with downregulated LPCAT2, TLR3, and TGFB2, among other immune-associated genes. LPCAT2 has been shown to regulate macrophage inflammatory gene expression with TLR genes [53]. The TGFB2 protein suppresses tumor growth by regulating cell growth, proliferation, and apoptosis. The downregulation of these IA genes suggest that these *D. acidovorans*, *G. vaginalis*, and *N. hamburgensisX14* promote tumor progression in PC by actively suppressing immune cell expression, instead of increasing inflammation. 

#### 2.6.3. Correlation to Immune Cell Types

Using CIBERSORTx software, we found the strongest correlation to exist between PC microbes and regulatory T-cells, with a weighted sum of −log(*p*-value) equal to 7. Regulatory T-cells weaken the immune system by suppressing T-cell expression. This result is consistent with microbe correlations to downregulated IA genes that regulate immune cell expression. We also observed statistically significant correlations between PC microbes and natural killer (NK) cells, M1 macrophages, and M2 macrophages. Interestingly, we found all correlations between microbe abundance and immune cell expression to be positive (Figure 4C). While positive correlations to NK and macrophage cell expression is unexpected, we explored explanations for this in the Discussion section. 

### 2.7. Correlation to AR Expression

**Figure 5:**
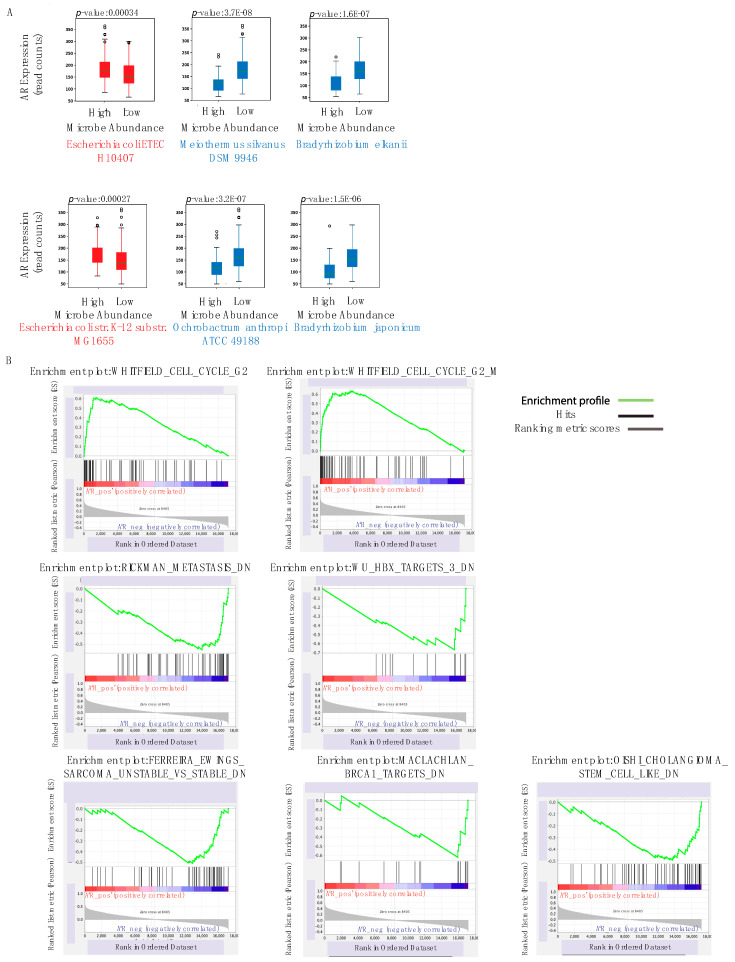
(**A**) Select boxplots of microbes that are most significantly correlated with Androgen Receptor (AR) expression. Microbe abundance was converted to a binary variable of “High” and “Low” relative to median abundance across PC patients and is represented by the x-axis. The y-axis represents AR expression, which was defined as a continuous variable of read counts. Red boxplots show microbes that are positively correlated with high AR expression and blue boxplots show microbes that are negatively correlated with high AR expression. (**B**) Gene sets and immunologic signatures that are most correlated with AR expression, identified by Gene-set Enrichment Analysis (GSEA). Whitfield_Cell_Cycle_G2 gene sets were found to be positively correlated with AR expression, and the signatures below were found to be negatively correlated with AR expression.

Among the 230 microbes that are significantly negatively correlated with AR expression (*p* < 0.05), we identified *Bradyrhizobium elkanii, Ochrobactrum anthropi ATCC 49188*, and *Bradyrhizobium japonicum* to have the strongest negative association (Figure 5A). We also found two microbes *Escherichia coli ETEC H10407* and *Escherichia coli str. K-12 substr. MG1655* to be significantly positively correlated with AR expression (Figure 5A). *E. coli* strains are known prostatitis-related microbes, which is consistent with their abundance being positively correlated with increased AR expression [54]. 

By conducting GSEA, we correlated curated gene sets and immunologic signatures with AR expression to investigate potential mechanisms by which these microbes may regulate the AR (Figure 5B). We found that the Whitfield Cell Cycle G2 and the Whitfield Cell Cycle G2 M pathways are positively associated with AR expression. The Whitfield Cell Cycle G2 pathway describes genes that are expressed during the G2 phase of the cell cycle [55]. The upregulation of this pathway may contribute to increased proliferation and tumor growth, which is consistent with the established finding that increased AR expression is characteristic of PC patients. Increased AR expression was also correlated with a number of dysregulated gene signatures in other cancers: genes down-regulated in metastatic versus non-metastatic head and neck squamous cell carcinoma samples, genes down-regulated by the expression of HBV X protein, genes down-regulated in genomically unstable Ewing’s sarcoma tumors compared to the stable ones, genes down-regulated in SW480 cells (colon cancer with mutated p53 upon expression of BRCA1 off an adenovirus vector), and genes under-expressed in stem cell-like cholangiocellular carcinoma. Given that increased AR expression has been associated with poorer prognosis in some of these cancers (early-stage hepatocellular carcinoma and colon cancer), these results suggest that these pathways may serve as effective therapeutic targets across multiple cancers, including head and neck cancer and breast cancer [56,57]. However, further research is necessary to conclude how these pathways that directly regulate the AR are influenced by specific microbe species. This future research can lead to the development of probiotic treatments that can target and regulate pathway expression in cancer. 

### 2.8. Correlation to Stem-Cell Genes

**Figure 6:**
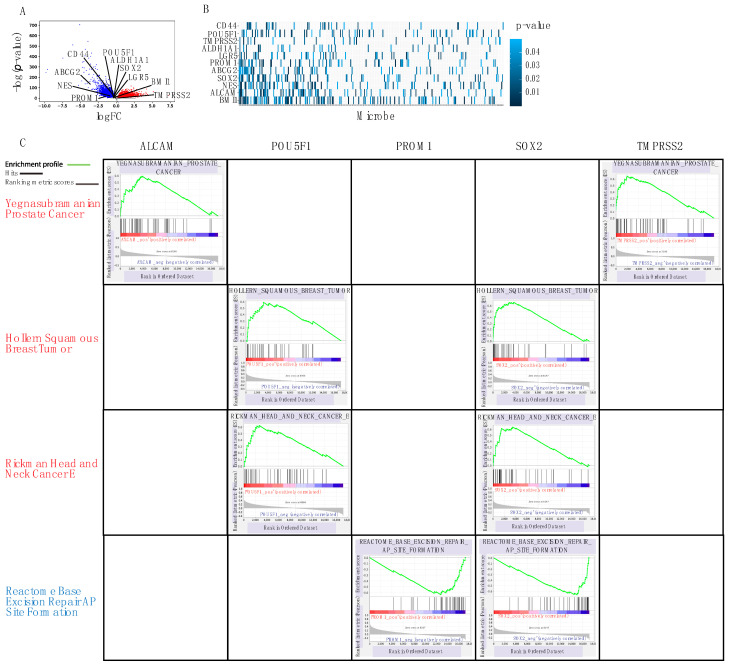
(**A**) Volcano plot showing significantly differentially expressed stem cell genes (ABCG2, ALDH1A1, BMI1, CD44, LGR5, NES, POU5F1, PROM1, SOX2, and TMPRSS2) relative to all genes. (**B**) Heatmap visualizing correlation between microbe expression and stem cell gene expression. Gradient of blue indicates strength of correlation, with dark blue associated with the strongest correlation. (**C**) Using GSEA analysis, we found the gene sets most correlated with the greatest number of stem cell genes. Yegnasubramanian_Prostate_Cancer, Hollern_Squamous_Breast_Tumor, and Rickman_Head_And_Neck_Cancer_E are positively correlated with stem-cell gene expression, and the Reactome_Base_Excision_Repair_AP_Site_Formation gene set is negatively correlated with stem-cell gene expression.

We investigated the following PCSCs: ABCG2, ALCAM, ALDH1A1, BMI1, CD44, LGR5, NES, POU5F1, PROM1, TMPRSS2, and SOX2 [58]. While only ABCG2 and PROM1 showed significant log-squared fold change, all 11 genes were found to be significantly dysregulated in PC samples (*p*-value < 0.05) (Figure 6A). Prior research also elucidated strong correlations between the expression and function of these genes and progression of prostate cancer. 

After correlating microbe expression with stem-cell gene expression, we found that *Staphylococcus aureus subsp. aureus MW2*, *Paraburkholderia phymatum STM815*, *Pseudomonas putida F1*, and *Haemophilus parainfluenzae T3T1* were correlated with the highest number of stem cell genes (Figure 6B). 

We found that the Yegnasubramanian Prostate Cancer, Hollern Squamous Breast Tumor, Rickman Head and Neck Cancer E gene sets are positively correlated with stem cell gene expression. The Yegnasubramanian Prostate Cancer gene set is positively correlated with ALCAM and TMPRSS2 gene expression. The Yegnasubramanian Prostate Cancer gene set consists of genes expressed in prostate cancer cell lines, but not in normal prostate epithelial cells or stromal cells. The cancer testis antigen genes in the Yegnasubramanian gene set undergo the hypomethylation of CpG (CG site) dinucleotides in genomic DNA and are overexpressed in metastatic prostate cancers. This is an epigenetic alteration that may directly result from Staphylococcus aureus subsp. aureus MW2 and other specific microbes that inhabit the PC tumor. The Hollern Squamous Breast Tumor gene set is positively correlated with POU5F1 and SOX2 gene expression and consists of genes that have high expression in mammary tumors of squamous epithelium histology [59]. The Rickman Head and Neck Cancer gene set is positively correlated with POU5F1 and SOX2 gene expression and consists of genes identifying an intrinsic group in head and neck squamous cell carcinoma (HNSCC) [60]. Since the Rickman Head and Neck Cancer E gene set describes genes with metastatic-related functions, the correlation between microbial presence and the overexpression of these genes suggests that specific microbes play an important role in determining the development of metastases and the aggressiveness of PC. The Reactome Base Excision Repair AP Site Formation gene set is negatively correlated with PROM1 and SOX2 gene expression. It consists of genes that are responsible for base excision repair initiated by DNA glycosylases (Figure 6C) [61], suggesting that microbes contribute to PC progression by inhibiting repair pathways, such as the Reactome Base Excision Repair AP Site Formation gene set, leading to uncontrolled cancer cell growth. 

### 2.9. Qiime Diversity Calculations 

**Figure 7:**
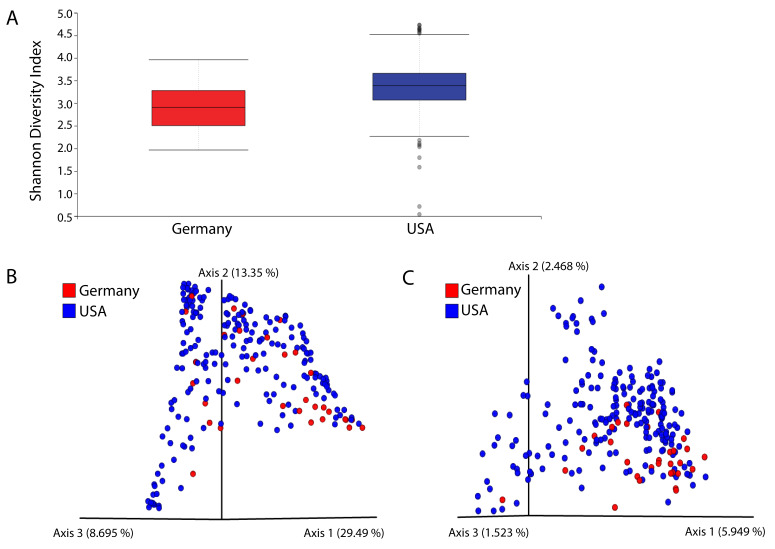
(**A**) Qiime boxplots showing that patients from the U.S. had a higher Shannon Diversity Index than patients from Germany. (**B**) Principal Component Analysis Emperor plot based on Bray–Curtis diversity metric. (**C**) PCoA Emperor plot based on Jaccard diversity metric.

We observed that TCGA data included PC patients exclusively from the United States and Germany. Taking into account that residing in these different regions may have a significant impact on microbiome composition, we conducted Qiime diversity calculations on PC patient samples. We found that samples from patients in the U.S. had a higher Shannon Diversity Index, which measures the abundance and evenness of the species present, than samples from patients in Germany (Figure 7A). Overall, we concluded that there is no significant difference in diversity between patients of the two countries after we measured diversity using numerous metrics (Figure 7B,C). 

### 2.10. Validation with Kim Dataset

**Figure 8:**
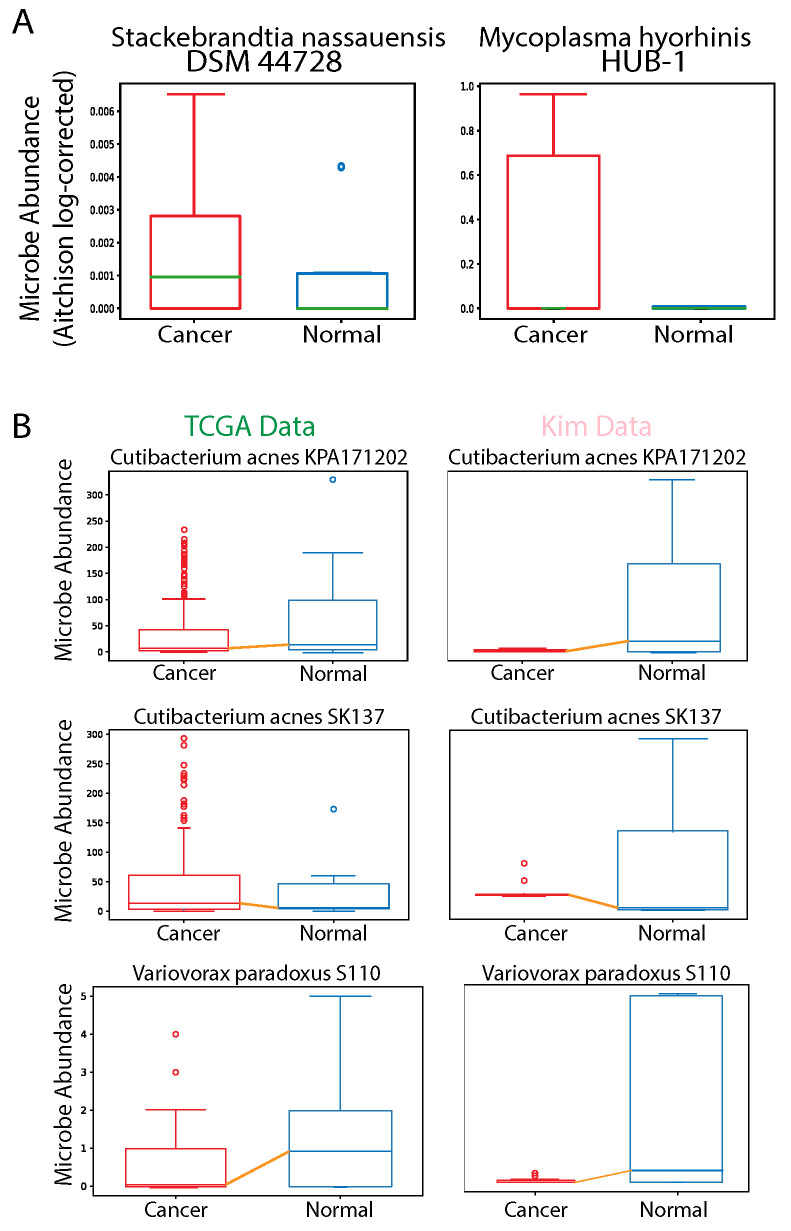
(**A**) Boxplots illustrating differential abundance of pro-tumor microbes in prostate cancer cell data, which were obtained from the study published by JH Kim et al [62]. (**B**) Boxplots of microbes that are similarly differentially abundant in the The Cancer Genome Atlas (TCGA) dataset and the Kim dataset (*p* < 0.05). Select boxplots illustrated based on lowest *p*-value.

We validated our findings on PRAD TCGA data by conducting differential abundance analysis (*t*-test, *p* < 0.05) and taxonomy group comparison analysis on prostate cancer cell and tissue and normal data obtained from a study published by Kim et. al [62]. Since the dataset used for validation had a very small sample size, we did not expect the results to comprehensively reflect what we had found from our much larger dataset obtained from TCGA. In addition, depth of sequencing and base pairs per read may have affected Pathoscope alignment processing. However, we found 11 differentially abundant microbes in the Kim dataset to overlap with our findings from differential abundance analysis in TCGA data (*p* < 0.05) (Figure 8A,B). We found evidence of additional pro-tumor microbes that may play a key role in prostate cancer progression. *Stackebrandtia nassauensis DSM 44728* was significantly upregulated in prostate cancer samples and has previously been found to produce uricase, an enzyme that breaks down anti-tumor uric acid [63]. Uric acid is a powerful antioxidant that protects against radical-caused aging and cancer. We also found *Mycoplasma hyorhinis HUB-1* to be significantly upregulated in prostate cancer samples, and it has been linked to tumorigenesis in gastric and prostate cancer, by increasing the expression of inflammatory and phosphorylating factors to increase cancer cell invasiveness [64,65,66]. 

## 3. Discussion

In this study, we conducted a comprehensive investigation of microbe abundance and its influence on the immune landscape and the development of prostate cancer. We measured the correlation of microbes with clinical variables including the Gleason score, TNM staging, and PSA values to observe associations between microbes and prostate cancer aggressiveness. We found that most of these microbes had pro-tumor or anti-tumor roles consistent with existing research, or they had not previously been identified in clinical cases. 

Of the four microbes we identified as negatively correlated with Gleason scores, *Pediococcus pentosaceus, Listeria monocytogenes*, and *Lactobacillus crispatus* have previously been shown to have anticancer roles. *Pediococcus pentosaceus* was an effective target for probiotic treatment in colon cancer, and *Listeria monocytogenes* is used in cancer immunotherapy [35,36]. These results suggest that these microbes have therapeutic potential in PC treatment as well. 

Of the microbes we identified as negatively correlated with TNM staging, *Methylobacterium* species are opportunistic pathogens found to infect immunocompromised hosts, and *M. radiotolerans* infections are common in leukemia and neutropenic cancer patients [44]. *Stenotrophomonas maltophilia* is a multidrug-resistant global opportunistic pathogen and frequently co-colonizes with *Pseudomonas aeruginosa.* The cell-to-cell communication between these bacterial species has shown to be a critical target for developing novel pharmacological therapies [67]. *M. silvanus* was also found to be differentially abundant in reformed smokers with lung adenocarcinoma [68]. While further research is necessary to explain these microbes’ negative correlation with TNM stage, our results show that the abundance of these microbes in PC tumors is unique, and therefore, their presence can be used as a diagnostic tool for PC. 

Examining microbes correlated with PSA levels, *C. concisus* is associated with chronic intestinal disease, and its emergence as an intestinal pathogen is believed to provide great insights on host-immune response and explain the heterogeneity of specific bacterial infections [69,70]. The unexpected positive correlation between *Thermus thermophilus* and PSA value suggests that *Thermus thermophilus*, and other microbe species, may play different roles in different cancers, based on their abundance, the tumor microenvironment, and other regulatory factors. The presence of *Streptococcus pneumoniae SPN032672* is positively correlated with inflammation and cytotoxicity, which is consistent with the microbe’s positive correlation with PSA levels [71]. *Xanthomonas albilineans GPE PC73*, *Herminiimonas arsenicoxydans*, and *Pseudarthrobacter chlorophenolicus A6*, all of which were strongly negatively correlated with PSA value, have not been previously identified in humans. 

In all, we have identified a collection of microbes with potential diagnostic and therapeutic uses for PC. Further research must be done to clarify the roles of microbes that have previously not been identified in clinical cases. 

By analyzing microbe influence on immune processes, we found that differentially abundant microbes may directly contribute to cancer development by increasing the rate of infection and actively suppressing T-cell activity. We found microbe abundance in PC tissue to be highly correlated with expression of regulatory T-cells, which suppress the activation and proliferation of effector T-cells and weaken the immune system. However, significant correlations between microbe expression and other immune cells indicate that anti-tumor microbes, such as *Listeria monocytogenes*, that are negatively correlated with PC phenotypes may have a significant role in recruiting immune cells to trigger an immune response. While no previous studies have shown *D. acidovorans SPH-1* in context of cancer, its differential abundance in immunocompromised and immunocompetent patients was found to be a significant contributor to sepsis [33]. *S. aureus* has been previously shown to cause prostate abscess, and *G. vaginalis* is abundant in numerous patients with urinary and prostate-related diseases [72]. Although no prior research has specifically highlighted these microbes’ role in prostate cancer, the differential abundance of these microbes in prostate cancer likely has a synergistic effect in inducing inflammation and potentially increasing the dysregulation of immune-associated genes, PCSC genes, and AR, ultimately increasing the rate of infection. 

Since high androgen levels are characteristic of PC, we investigated microbes’ role on the Androgen Receptor gene. *Bradyrhizobium elkanii*, *Ochrobactrum anthropi ATCC 49188,* and *Bradyrhizobium japonicum*, all negatively correlated with AR, may be tumor-suppressing microbes, while *Escherichia coli ETEC H10407* and *Escherichia coli str. K-12 substr. MG1655* make up the PC-promoting microbiome. By conducting GSEA analysis, we confirmed that increased AR expression directly relates to pathways associated with uncontrolled cell cycle regulation. 

Finally, we investigated microbes’ association with stem cell genes, as stem cell gene expression is a hallmark of metastatic cancer. We found that the *Staphylococcus aureus subsp. aureus MW2*, *Paraburkholderia phymatum STM815*, *Pseudomonas putida F1*, and *Haemophilus parainfluenzae T3T1* were correlated with the greatest number of stem cell genes. From GSEA pathway analysis, we found that PCSC’s primarily drive metastatic growth through cancer-promoting pathways, including Yegnasubramanian Prostate Cancer, Hollern Squamous Breast Tumor, and Rickman Head and Neck Cancer E gene sets. Based on these pathways, we believe microbes can contribute to the inhibition of DNA repair pathways, leading to uncontrolled cell proliferation and the development of metastases. 

Finally, we validated our findings with a small dataset in a study published by Kim et al [62]. We found 11 of the microbes that we identified to be most significantly differentially abundant to overlap among the datasets, and we identified *Stackebrandtia nassauensis DSM 44728* and *Mycoplasma hyorhinis HUB-1* to be important pro-tumor microbes. 

Since the microbes we identified were more abundant in PC samples than in normal samples, we would normally expect that these microbes promote cancer growth. However, as many of the microbes we specifically studied appear to have anti-tumor properties, we believe it is possible that the prostate cancer microenvironment is conducive for microbial growth. This in turn would promote the translocation of microbes from the gut and possibly from other locations to the prostate, as they would hone to tumors with a rich blood supply and relatively leaky aberrant vasculature. After infiltrating the tumor microenvironment, anti-tumor microbes may directly reduce cancer growth by triggering an immune response or may indirectly mitigate tumor growth by outcompeting cancer cells. However, further research is necessary to warrant the individual effects of anti-tumor microbes that are overabundant in PC. 

To the best of our knowledge, we are the first to use TCGA microbial reads to comprehensively describe the influence of the intratumor microbiome on various PC phenotypes of unknown causes, including elevated PSA levels, AR expression, stem-cell gene expression, and commonly dysregulated genes. While recently published studies using data obtained from TCGA have employed machine learning algorithms to identify microbial blood signatures as a cancer diagnostic tool, our study elucidates the roles of specific microbes in PC. We show how specific intratumor microbe species may play a pro-tumor or anti-tumor role in PC and can be used as specific targets in prebiotic or probiotic treatments. Our results are critical for guiding future research in studying more exact mechanisms for how microbes influence PC development and if these mechanisms can be manipulated to treat metastatic PC. 

## 4. Materials and Methods

### 4.1. Data Acquisition from TCGA

Raw whole-transcriptome RNA-sequencing data for tumor tissue were downloaded from the TCGA legacy archive for 242 PRAD patients. Detailed patient characteristics and clinical information can be found in Supplementary Table S2. According to the Supplemental Experimental Procedures provided by TCGA on The Molecular Taxonomy of Primary Prostate Cancer, all samples were were collected from patients diagnosed with prostate adenocarcinoma who had not received prior treatment (chemotherapy, radiotherapy, or hormonal ablation therapy). Each frozen primary tumor specimen was accompanied with an adjacent normal tissue specimen, and the specimens were shipped overnight from using a cryoport that maintained an average temperature of less than −180 °C. Hematoxylin and eosin-stained sections from tumor and normal tissue samples were independently reviewed to validate that the tumor specimen was histologically consistent with the allowable prostate adenocarcinoma subtypes. The RNA-sequencing data for adjacent solid normal tissue samples of 52 PRAD patients were also obtained. Adjacent normal specimens were also reviewed to ensure that no normal sample contained tumor cells. Level 3 normalized mRNA expression read counts for the above samples were downloaded from the Genomic Data Commons (GDC) data portal. Clinical information for all patients were downloaded from the Broad Genome Data Analysis Center (GDAC) Firehose. Genomic alteration information for each patient was obtained from the last analysis report (2016) of the Broad Institute TCGA Genome Data Analysis Center. 

### 4.2. Differential Microbial Abundance between Cancer and Normal Patients

Differential abundance analysis was conducted to compare microbe abundance (percent abundance) in cancer tissues to microbe abundance in normal tissues. Microbes present in less than 10 patients were excluded. Then, the Kruskal–Wallis analysis test was applied to determine differential abundance (*p* < 0.05). To validate our findings in the unpaired normal and cancer tissue samples, we conducted a paired t-test with the available adjacent tumor and solid tissue samples. We found 13 microbes that were differently distributed across normal and adjacent tumor samples (*p* < 0.05). All these microbes were also differentially abundant in our initial unpaired data analysis. 

### 4.3. Evaluation of Contamination Using Plates, Flow Cells, and Date of Sequencing

The abundance values of microbes were associated with plates on which the samples were stored prior to sequencing using the Kruskal–Wallis test. The abundance values were also plotted to the flow cells on which the samples were sequenced and subjected to the same examination. Finally, the flow cells were plotted in order of date sequenced on the boxplot. We observed that groups of flow cells sequenced near the same date would have a similar overexpression of specific bacterial species. Therefore, we applied a heuristic algorithm to extract the flow cell ranges where this overexpression occurs, which allowed us to determine potential contaminants’ relationship with the sequencing date. 

#### 4.3.1. Read Counts from Sequencing

Slope and R squared values were calculated for each individual microbe and plotted. Each microbe with a significantly large absolute value of slope was plotted individually. A higher magnitude of slope indicates that microbe expression is consistent throughout all samples from different patients, which would only be the case if the microbe is a contaminant. This method elicited zero microbial contaminants (Figure S1A). 

#### 4.3.2. Aligning Microbe Abundance by Date Sequenced

In this method, the abundance of microbes was plotted against the corresponding sequencing date of the sample (Figure S1B). We observed that *Capnocytophaga canimorsus*, *Propionigenium maris*, *Xanthomonas citri*, *Agrobacterium*, *Mycoplasma mycoides*, and *Cellvibrio japonicus Ueda 107* have abnormal peaks on different days, which indicates the probable contamination of the specific microbe on a specific day. Among these, *Agrobacterium* and *Propionigenium maris* are known as agents of nosocomial infections [32,33,34]. 

### 4.4. Correlation of Microbial Abundance to Clinical Variables

Clinical variable analyses were performed using the Kruskal–Wallis test. Microbe abundance was converted into a binary variable of “High” and “Low” based on presence or absence of microbes in tumor samples for correlations with Gleason score and PSA level. We did not make a distinction between high and low Gleason score and PSA level, but we defined them as continuous variables. Univariate Cox regression analysis was used to identify candidates that were significantly associated with the following clinical variables: Gleason score and PSA level (*p* < 0.05). For correlation between microbe abundance and TNM stage, microbe abundance data were defined as a continuous variable of counts. 

### 4.5. Probability of Correlation Overlap

We also found microbes that were correlated with multiple clinical variables, including Gleason Score, PSA level, and AR. These were *Stenotrophomonas maltophilia*, *Anoxybacillus flavithermus WK1*, *Mesorhizobium huakuii*, *Bradyrhizobium japonicum*, *Listeria monocytogenes*, and *Comamonas testosteroni*. Traditionally, it has been accepted that the Gleason score would correlate with TNM, and with that belief, it would be logical to assume that all of the microbes implicated in these various clinical variables would be the same [73]. However, there are conflicting reports on correlations and a growing amount of evidence that suggests that this assumption may not necessarily be true. A study of 205 patients that showed that the serum total PSA at presentation (iPSA) was not significantly correlated with Gleason score [74]. This is further supported by a prior Japanese study, which investigated pathologic outcomes of over 400 men with low PSA levels and prostate cancer; the authors found that pathologic outcomes were significantly worse among men with a PSA level of less than 3.5, compared with men with a PSA level between 3.5 and 10 [75]. These show that PSA level and Gleason score are not definitively correlated, and they may even have unexpected directions of correlation at low PSA levels. A study of patients with untreated prostate cancer also found that the percentage of free PSA was not correlated with TNM stage [76]. Furthermore, there is also a lack of correlation between Gleason score and TNM staging, as a study found little overlap between advanced prostate cancer cases defined by these two methods [77]. Current research also shows that there is no correlation between androgen levels and PSA levels in prostate cancer patients [78]. In all, these studies suggest that the lack of very rigorous correlation among these clinical variables is uncertain and could explain why most of the microbes that we found to correlate with multiple clinical variables were weakly correlated.

In addition, we calculated the probability that the clinical variables are in fact associated with each other, given their correlations to specific microbes that we identified. In order to eliminate confusion in our calculations, we converted all measurements of clinical variables and microbe abundance into binary variables. We created a contingency table to determine the chi squared statistic and find the probability that the abundance of a specific microbe correlated with one clinical variable, such as AR expression, given the correlation between the abundance of that same microbe with another clinical variable, such as PSA level. This calculation gave us the probability of overlap between two clinical variables. In order to calculate the probability for overlaps to occur across all four clinical variables, we modeled our problem as a binomial distribution:

(1) $$f\left(k,n,p\right)=PrPr\;\left(k;n,p\right)\;=PrPr\;\left(X=k\right)\;=\left(\frac{n}{k}\right)p^{k}{\left(1-p\right)}^{n-k},$$

where *k* represents the number of microbes we expect to observe overlap in, *n* represents the total number of microbes, and *p* represents the probability that a randomly chosen sample would have overlap, which is equal to the intersection of the correlations between microbe abundance with two clinical variables. For most cases, we could not derive an exact solution for each microbe, since each term in the expression would approach zero. Thus, we could infer that the summation of probabilities from all microbe cases would be equal to zero, given that the probability of each microbe case is close to zero. For some cases, we were able to estimate the total probability of overlaps in microbe correlations across multiple clinical variables by summing the terms with *n* and *k* values in each binomial expression that resulted in a nonzero result. We found small, nonzero estimations for the probability of overlap between AR expression and PSA level, between AR expression and Gleason score, and between PSA level and Gleason score for *Listeria monocytogenes*, although in our study, we primarily investigated *Lm* and its strong correlation with Gleason score. This proof agrees with prior research and our reasoning that the correlation between any two clinical variables, such as high AR expression and elevated PSA level, may not necessarily be strong, and it is not definitively clear. Therefore, we decided to investigate microbes that most strongly correlated with one clinical variable (according to lowest *p*-value), instead of studying microbes we found that were more weakly correlated with multiple clinical variables. While some of the previously listed overlapping microbes, which correlate with multiple clinical variables, may be important as they are associated with multiple clinical variables, in vitro and in vivo experiments are needed to validate their relevance and significance since their strength of correlation, as determined by *p*-value, was lower than those of the microbes we specifically discussed.

### 4.6. Correlation of Microbial Abundance with Genomic Alterations Using REVEALER

The Repeated Evaluation of Variables conditionAL Entropy and Redundancy (REVEALER) program was used to identify a statistically significant association of genomic alterations (amplifications, deletions, or mutations) with the abundance of individual microbes. We defined an association as significant if the absolute value of its Conditional Information Coefficient (CIC) value was greater than 0.3. A Circos plot was constructed using data from the REVEALER plots to map the genomic region of each genomic alteration that significantly correlated with microbe abundance. 

### 4.7. Correlation of Microbial Abundance with IA Gene Expression

We constructed a second Circos plot to map all IA genes found to be significantly correlated with REVEALER-associated microbes. Strength of association was determined using edgeR, and differential expression analysis was performed between mRNA expression of PC and normal tissues to identify significantly dysregulated IA genes (FDR < 0.05 and |log fold change| > 1). The Kruskal–Wallis test was used to correlate the abundance of significantly dysregulated microbes to significantly dysregulated IA genes (*p* < 0.05). Microbe abundance was modeled as a binary variable of presence and absence. 

### 4.8. Correlation of Microbial Abundance to Immune Cell Types

Using the software tool CIBERSORTx, we deconvoluted the tumor-infiltrating lymphocyte population from microbial abundance data. The expression of 22 types of tumor infiltrating lymphocytes were analyzed: naïve B-cells, memory B-cells, plasma cells, CD8 T-cells, CD4 naïve T-cells, CD4 memory resting T-cells, CD4 memory-activated T-cells, follicular helper T-cells, regulatory T-cells, gamma-delta T-cells, resting NK cells, activated NK cells, monocytes, M0–M2 macrophages, resting dendritic cells, activated dendritic cells, resting mast cells, activated mast cells, eosinophils, and neutrophils. The Kruskal–Wallis test was employed to correlate immune cell expression levels with microbial abundance (*p* < 0.05). Microbe abundance was modeled as a binary variable of presence and absence.

### 4.9. Correlation of Microbial Abundance to Expression of Immune-Associated Genes, AR, and Stem-Cell Genes

Differential expression analysis between mRNA expression of PC and normal tissues was conducted using edgeR. The cutoff, false discovery rate (FDR) < 0.05 and |log fold change| > 1, was used to identify significantly dysregulated genes. Microbe abundance was modeled as a binary variable of presence and absence, and the Kruskal–Wallis test was employed to correlate the abundance of significantly dysregulated microbes to significantly dysregulated genes (*p* < 0.05). Microbe abundance was converted into a binary variable of “High” and “Low” based on the presence or absence of microbes in tumor samples for correlations with AR expression. We did not make a distinction between high and low AR expression, but we defined AR expression as a continuous variable. 

### 4.10. Correlation of Microbial Abundance with Immune Pathway Using GSEA

GSEA was used to identify biological pathways and signatures associated with AR and stem-cell gene expression. Known gene sets were obtained from the Molecular Signature Database (MSigDB), and canonical pathways (C2) and immunologic signatures (C7) were examined. Using Pearson’s correlation for the continuous phenotypes and signal-to-noise ratio for categorical phenotypes, AR expression and stem cell gene expression were correlated to the above gene sets to generate enrichment scores and statistical significance values.

### 4.11. Qiime2 Diversity Calculations

The Qiime2 microbiome bioinformatics platform was used to compare alpha and beta diversities of patients from the U.S. and Germany. The Shannon Diversity Index was used to evaluate the richness and evenness of the microbiota of each country’s samples, while Bray–Curtis and Jaccard distances were used to compare the similarities between samples of the two countries. 

## 5. Conclusions

In summary, our study provides key insights on specific microbes and their influence in PC tumors. We identified microbes that are differentially abundant in PC, and we measured correlations between microbial abundance and established PC risk factors to show how microbes may regulate tumor development via cancer-immune interactions (Table S1). We found that specific microbes, such as *Listeria monocytogenes*, *Lactobacillus crispatus*, and *Thermus thermophilus*, mitigate tumor growth, while other microbes, such as *Nevskia ramosa* and *S. aureus*, are cancer-promoting, by inducing inflammation, promoting immunosuppression, and regulating expression of PCSCs that drive metastases. Future research using higher resolution bacterial sequencing techniques, more rigorous methods of contamination correction, and in vitro and in vivo experiments are necessary to validate our current findings on the intratumor microbiome and its influence in PC. 

## Supplementary Materials

The following are available online at https://www.mdpi.com/2072-6694/12/9/2524/s1, Figure S1: (**A**) Microbial contamination correction with sequencing read counts. R-squared and slope was calculated to detect contaminants, microbes with large slopes. (**B**) Contamination correction by date sequenced, Table S1: Microbes in PC. Columns “Role in PC” and “Correlation” are written according to results found in this study. Column “Known Function” is written according to prior research, Table S2: Detailed patient characteristics and clinical information.
